# Macro roles for microRNAs in neurodegenerative diseases

**DOI:** 10.1016/j.ncrna.2018.07.001

**Published:** 2018-07-31

**Authors:** Dipen Rajgor

**Affiliations:** Department of Pharmacology, University of Colorado Denver School of Medicine, Aurora, CO, 80045, USA

## Abstract

Neurodegenerative diseases (NDs) are typically adult-onset progressive disorders that perturb neuronal function, plasticity and health that arise through a host of one or more genetic and/or environmental factors. Over the last decade, numerous studies have shown that mutations in RNA binding proteins and changes in miRNA profiles within the brain are significantly altered during the progression towards NDs – suggesting miRNAs may be one of these contributing factors. Interestingly, the molecular and cellular functions of miRNAs in NDs is largely understudied and could remain a possible avenue for exploring therapeutic treatments for various NDs. In this review, I describe findings which have implicated miRNAs in various NDs and discuss how future studies focused around miRNA-mediated gene silencing could aid in furthering our understanding of maintaining a healthy brain.

## 1 Introduction

The mammalian brain is a complex structure, made up of millions of interlinked neuronal circuits that form through synaptic connections. During aging, perturbations of synaptic connections can lead to neuronal death and begin the initiation towards neurodegenerative diseases (NDs). NDs cover a broad spectrum of disorders associated with cognition and/or movement. The best studied and most common disorders include Parkinson's disease, Alzheimer's disease, Amyotrophic Lateral Sclerosis, Huntington's disease and the spinocerebellar ataxias [[1], [2], [3], [4], [5]]. In these diseases, genetic mutations resulting in protein aggregation is a common theme and perturbed mechanisms associated with pathogenesis involve multiple fundamental cellular pathways involving protein folding, protein clearance, RNA processing and metabolism processes [[6], [7], [8]]. Therefore, to fully understand the mechanisms underlying NDs, requires studying a wide range of cellular processes and machineries.

**Fig. 1:**
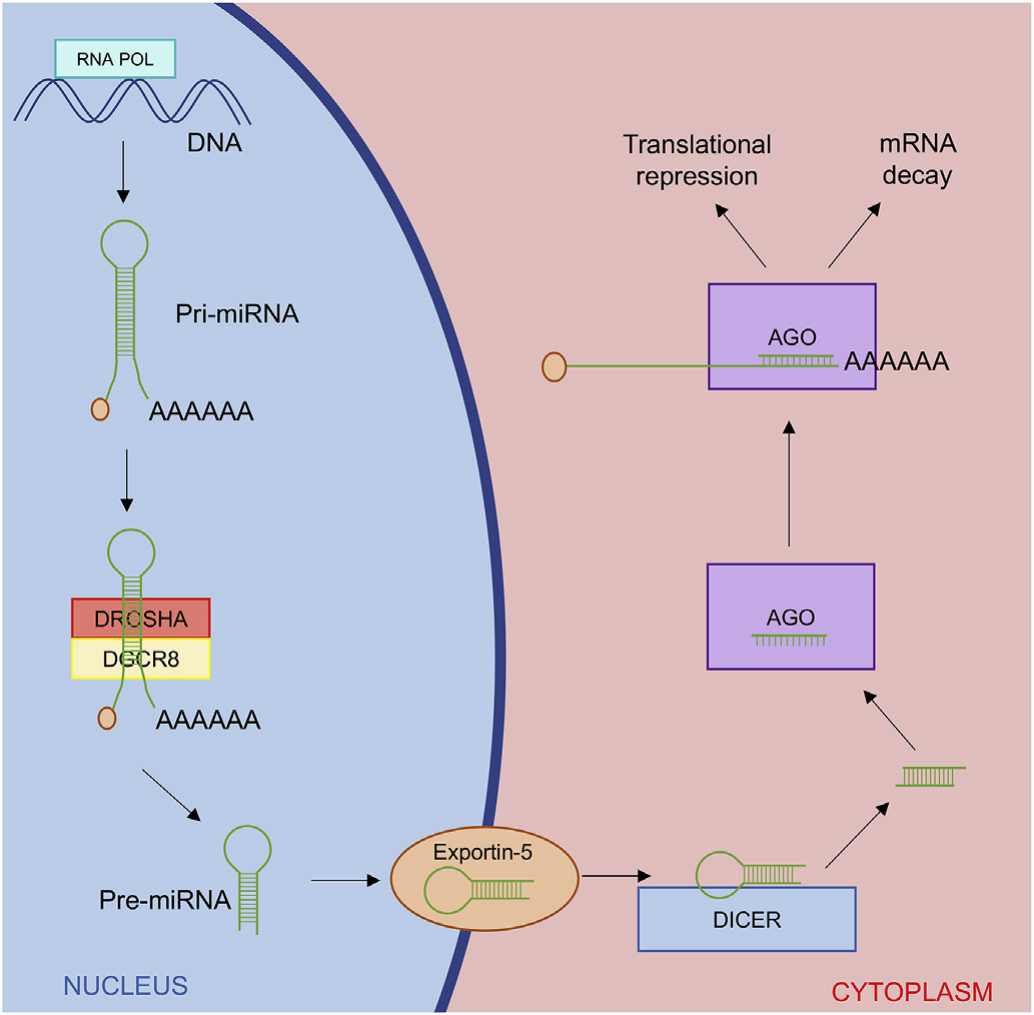
MiRNA biogenesis. miRNAs are transcribed from the genome by RNA polymerases II or III as primary-miRNA (Pri-miRNA). Pri-miRNAs are modified by a cap structure and polyadenylation. The pri-miRNA is processed in the nucleus by the Drosha/DGCR8 microprocessor complex which crops the pri-miRNA into a shorter hairpin-shaped precursor-miRNA (Pre-miRNA). The pre-miRNA is exported to cytoplasm via Exportin-5 and processed by Dicer which removes the hairpin. Next, one of the strands of the miRNA duplex is incorporated into Argonaute (AGO) proteins to form the miRNA inducing silencing complex (miRISC), which silences the mRNA via translational repression or by mRNA degradation.

miRNAs are a class of small non-coding RNAs that function in post-transcriptional gene expression. They are transcribed in the nucleus and subsequently cleaved by the Drosha and DGCR8 containing microprocessor complex (Fig. 1). The resulting pre-miRNA is exported to the cytosol and further processed by Dicer to an intermediate miRNA duplex. The leading miRNA strand is loaded into the miRNA-induced silencing complex (miRISC) and guided to target mRNAs to which it pairs with sequences primarily in the 3′ untranslated regions (UTRs) of the mRNA. This interaction leads to translational repression or degradation of the target mRNA [9]. Importantly, in neuronal dendrites Drosha is able to locally process pre-miRNAs locally at dendritic spines in an activity dependent manner which allows protein synthesis to be modulated at single synapses [10,11]. The unrestrained binding of miRNAs to target mRNAs allows a single class of miRNAs to repress multiple target transcripts involved in specific processes [12], which is an important feature for dictating synaptic plasticity by regulating local translation of dendritic transcripts [13].

The brain expresses more miRNAs than any other mammalian organ and therefore it is not surprising miRNAs have emerged as key regulators of neuronal development and plasticity [[13], [14], [15]]. Likewise, it is also not surprising that when miRNA regulation is hampered it leads to the onset of various NDs [16]. Therefore, NDs can be contemplated as RNA disorders in which the dysregulation of miRNAs or their binding proteins is a contributing factor in neurodegeneration [17].

In this review, I will summarize how miRNA expression is perturbed in various NDs and how this influences downstream pathways which further encourage cellular mechanisms leading to neuronal death.

### 1.1 miRNAs in Alzheimer's disease

Alzheimer's disease (AD) is the most common form of dementia that affects more than 20% of individuals over 80 years of age. AD is characteristically diagnosed by progressive memory loss and impairment of cognitive functions that prevent people from performing normal daily activities. Current therapeutic treatments only slow down progression of AD to a limited degree, which make identifying new potential biomarkers that could help in early detection of AD essential [18].

**Fig. 2:**
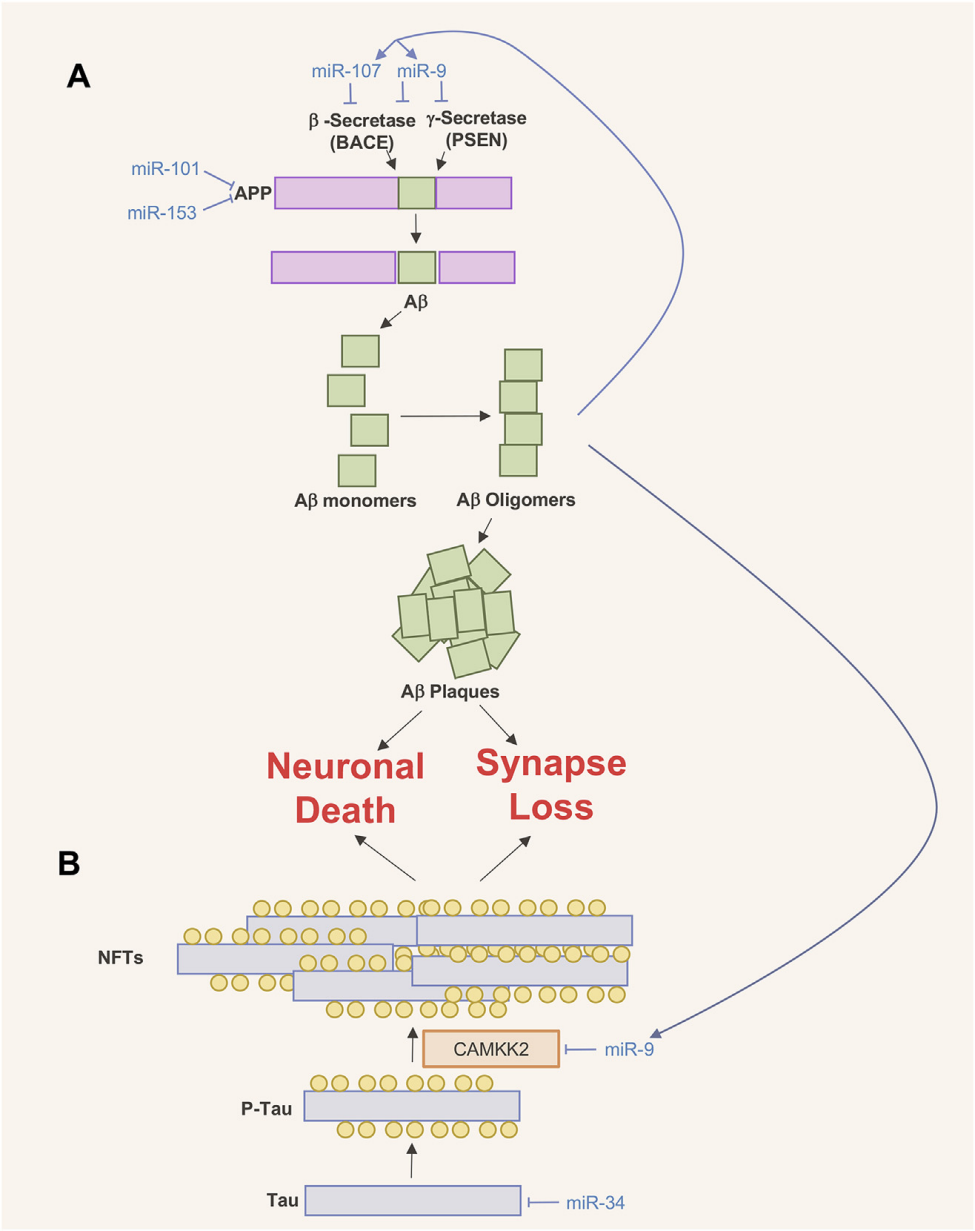
MiRNAs involved in the pathogenesis of Alzheimer's disease (AD). A) miRNAs which repress expression of APP or APP metabolizing enzymes are down-regulated in AD, leading to enhanced translation of BACE and PSEN. Additionally, Ab plaques further reduce expression of BACE and PSEN targeting miRNAs. miR-107, miR-9, miR-101 and miR-153 are shown as examples. B) miRNAs regulate Tau synthesis and formation of Neurofibrillary Tangles (NFTs). Both NFTs and Ab plaques contribute to the loss of synapses and neuronal death associated with AD. miR-9 and miR-34 are shown as examples.

Intracellular neurofibrillary tangles (NFT) formation and extracellular deposition of amyloid-β (Aβ) in the brain are the two major protein deformities leading to AD. NFTs come from abnormal aggregation of the hyperphosphorylated microtubule associated protein Tau, whereas Aβ peptides arise from the sequential cleavage of membrane-spanning amyloid precursor protein (APP) by the beta-secretase APP cleaving enzyme 1 (BACE1) and the γ-secretase complex containing the presenilin (PSEN) proteins in the catalytic domain (Fig. 2). Although mutations in *APP*, *PSEN1,* or *PSEN2* have been identified to pre-dominantly cause to AD, other mechanisms which lead to AD pathology are being investigated as potential contributors [19].

miRNA array experiments have demonstrated that AD brains exhibit significantly different miRNA profiles compared to healthy controls [[20], [21], [22]]. Over the last decade, a wide array of miRNAs targeting proteins involved in AD pathogenesis have been identified (Table 1).

**Table 1:** MiRNAs implicated in AD.

miRNA	Targets
miR-9	BACE1, PSEN1, SIRT1, CAMKK2
miR-29	BACE1, SPTLC2
miR-34	Tau, SIRT1, BCL2
miR-101	APP
miR-107	BACE1, ADAM10
miR-124	BACE1
miR-153	APP
miR-181	SIRT1, SPTLC1, BCL2, TRIM2
miR-195	BACE1

miR-9 is highly expressed in hippocampus, the region of the brain associated with memory and learning. miR-9 reduction in AD has been shown in various human AD brain samples, mouse models and neuronal cell culture models and is generally regarded as being neuroprotective [[23], [24], [25]]. miR-9 targets a number of proteins involved in AD pathogenesis pathways, including BACE1, PSEN1, Sirtuin-1 (a protein involved in reducing Aβ peptides and anti-aging) and Calcium/Calmodulin Dependent Protein Kinase Kinase 2 (CAMKK2) [[26], [27], [28]]. CAMKK2 is able to phosphorylate tau and its activity is enhanced in hippocampal neurons treated with Aβ peptides. Overactive CAMKK2 seen in response to Aβ stimulation contributes to dendritic spine loss and its signaling is therefore important in memory and learning that is hampered in AD [28]. Furthermore, hippocampal neurons overexpressing miR-9 are somewhat resistant to Aβ-mediated CAMKK2 Tau phosphorylation and dendritic spine loss [29]. Together these experiments demonstrate that miR-9 it is a strong therapeutic candidate for potentially preventing AD.

miR-107 is down-regulated in temporal cortex during early stage of AD [30]. It targets BACE1 and the metalloproteinase ADAM10 which is also involved in APP processing [31]. miR-107 has also been shown to target cofilin, an actin binding protein which dissembles actin filaments in dendritic spine heads and therefore important in memory and learning [32]. miR-107 expression has a negative correlation with NFT formation and together these factors suggest it is a major contributor in AD progression.

Many other miRNAs have been associated with AD which carry on with the theme of either directly or indirectly regulating the translation of proteins involved in AD pathogenesis (see review by *Miya Shaik* et al. for excellent detailed review [33]). Most miRNAs have implications in APP processing, neuroinflammation and tau phosphorylation, whilst others are involved in more than one of these processes, suggesting miRNAs may provide good therapeutic targets for interventions or treating a range of biological deformities associated with AD.

### 1.2 miRNAs in Parkinson's disease

Parkinson's disease (PD) is the second most common neurodegenerative disease after AD. Loss of dopaminergic neurons in the substantia nigra region of the brain leads to impairment of motor activities and decline in cognitive functions [34]. Symptoms of PD include tremors, slow movements, and poor balance. The neurotransmitter dopamine transmits messages to the substantia nigra which controls movement and coordination, and therefore depletion of dopamine levels leads to inhibition of motor functions resulting in movement difficulties [34].

**Fig. 3:**
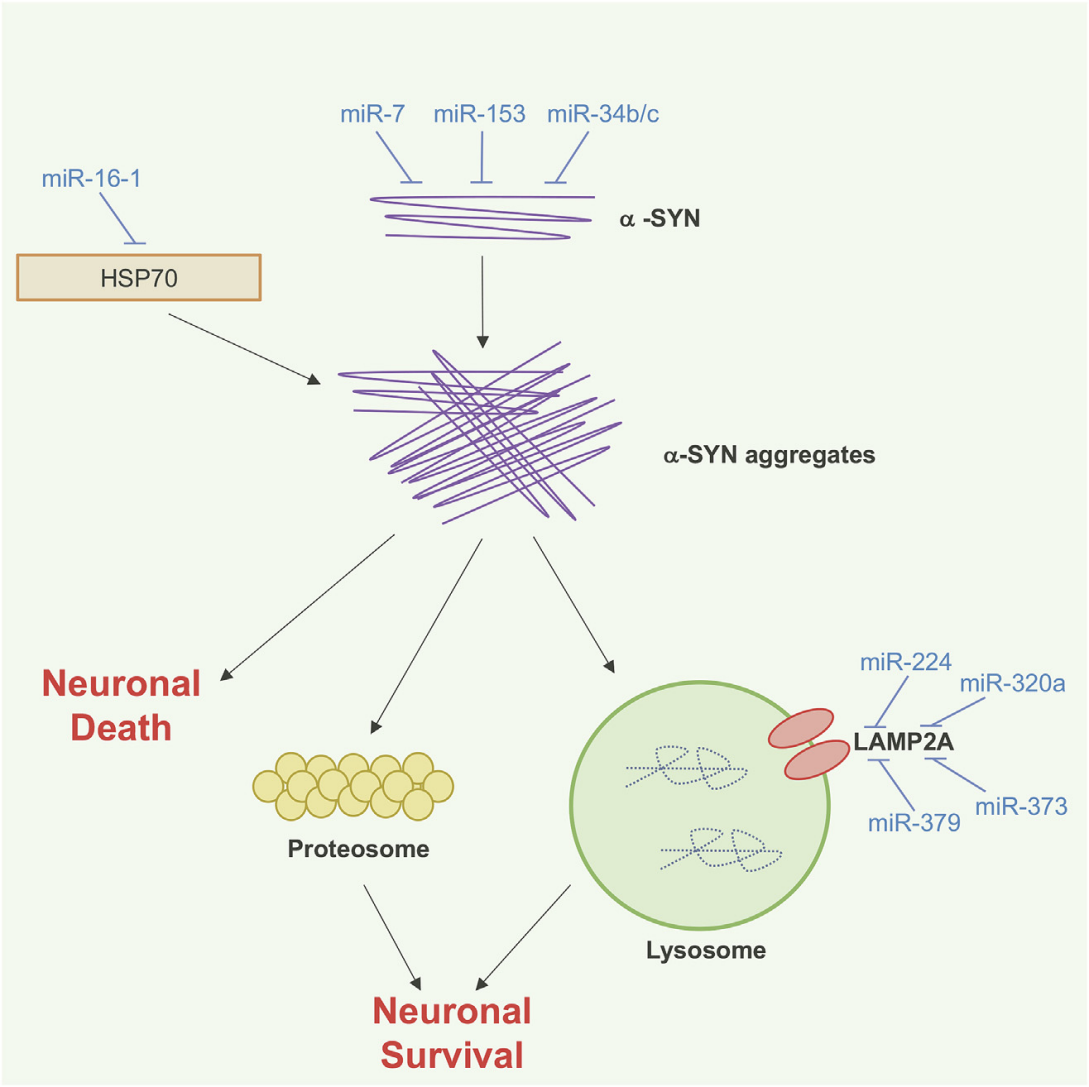
MiRNAs involved in the pathogenesis of Parkinson's disease (PD). miRNAs which repress expression of a-synuclein (a-SYN) are down-regulated in PD, leading to enhanced translation and aggregation of a-SYN. a-SYN aggregates are not cleared by normal cellular degradation machineries, often because miRNA-mediated silencing events which regulate translation of proteins involved in a-SYN clearance are also disrupted in PD. miR-7, miR-153 and miR-34 b/c target a-SYN. miR-16-1 targets HSP70. miR-224, miR-320a, miR-373 and miR-379 target LAMP2A.

Approximately 30% of PD cases are hereditary and usually caused by mutations in one of the following proteins: α-synuclein (α-SYN), Leucine-rich repeat kinase 2 (LRRK2), DJ-1, Parkin. α-SYN is involved in clustering synaptic vesicles at presynaptic terminals and mutations or gene duplication which promote α-SYN misfolding or overexpression leads to the onset of PD [[35], [36], [37]]. The cellular changes in PD due to α-SYN mutations or overexpression are illustrated by the presence of α-SYN containing cytoplasmic Lewy bodies [38,39]. A number of miRNAs are predicted to regulate α-SYN aggregation, either by directly targeting its repression or by acting indirectly through other proteins (Fig. 3, Table 2). miR-7, miR-153, miR-34 b and miR-34c are highly expressed in the brain and target the 3′UTR of *SCNA* encoding α-SYN [[40], [41], [42]]. Interestingly, levels of miR-34 b and miR-34c are downregulated in the brain of patients suffering from PD and polymorphism in the miR-34 b binding site in the 3′UTR of *SNCA* mRNA increase α-SYN protein levels. Inhibiting miR-34 b and miR-34c in neuroblastoma cells results in loss of mitochondrial membrane potential and elevates oxidative stress, further enhancing PD etiology [41]. Molecular chaperons, such as Heat Shock Proteins (HSPs), are important for maintaining homeostasis of proteins by facilitating protein folding, degradation and preventing protein aggregation [7]. Disruptions in HSPs are considered to play a key role in α-SYN aggregation in PD [43,44]. miR-16-1 represses HSP70 in cells overexpressing α-SYN and showed indirect regulation of α-SYN by increasing its expression [45]. Furthermore, miRNAs targeting lysosomal proteins such as lysosomal associated membrane protein 2 A (LAMP-2a) are also hampered in PD, which leads to aggregation of α-SYN. miR-224, miR- 320a, miR-373 and miR-379 are four 4 miRNAs which are predicted to target 3′UTR of LAMP-2A and are up-regulated in PD samples [46,47]. These miRNAs showed a dose-dependent decrease in endogenous LAMP-2A protein, resulting in significantly elevated levels of α-SYN accumulation [46].

**Table 2:** MiRNAs implicated in PD.

miRNA	Targets
miR-7	α-SYN
miR-16-1	HSP70
miR-34 b	α-SYN
miR-34c	α-SYN
miR-153	α-SYN
miR-138-2-3p	LRRK2
miR-205	LRRK2
miR-224	LAMP-2A
miR-320a	LAMP-2A
miR-373	LAMP-2A
miR-379	LAMP-2A
miR-4639-5p	DJ-1
miR-494	DJ-1

### 1.3 miRNAs in Huntington's disease

Huntington's disease (HD) is a genetic ND caused by abnormal expansion of polyglutamine (polyQ) repeats in the gene encoding the huntingtin (Htt) protein, which leads to the loss of medium spiny neurons in the striatum, progressive cognitive impairment, neuropsychiatric defects, and involuntary choreiform movements [48]. Htt interacts with the essential transcriptional repressor, Repressor Element 1 Silencing Transcription Factor (REST) in neurons [49]. WT huntingtin isolates REST in the soma of neurons, whereas mutant htt impedes this interaction resulting in nuclear accumulation of REST and increased altered transcription of neuronal miRNAs in HD [50]. Unlike AD and PD, the role of miRNAs in HD has not been extensively studied, however various different animal models suggest de-regulated miRNA-mediated gene regulation is a contributor to HD [51,52]. *Lee* et al. profiled miRNA expression and miRNA regulators in three different HD animal models [51]. They used two transgenic models of HD, YAC128 and R6/2 mice, and a 3-nitropropionic acid-induced striatal degeneration rat model. These animal models showed differential miRNA expressions throughout development, suggesting altered miRNA expression levels may vary extensively from case to case and show dynamic changes throughout development making it difficult to pinpoint miRNAs which may contribute to HD pathology. Interestingly, these animal models showed curious changes in the expression levels of proteins involved in miRNA function. For example in the YAC128 model, levels of DROSHA and DGCR8 were significantly elevated in 5 month aged mice, but not 12 month aged mice. This suggests miRNA biogenesis and function may be significantly hampered in early stages of HD. One study has also examined the miRNA expression profile in the frontal cortex of fetal and newborn HD monkeys [52]. They identified a 11 miRNAs to be significant dysregulated the HD monkeys and these miRNAs correlated with gene targets associated with HD canonical signaling pathways, including the Huntington protein itself and Huntingtin Interacting Protein 1. Interestingly, miR-124 has emerged as a miRNA which could be used to treat, or at least slowdown the progression of HD [53]. When this miR-124 is injected into a transgenic mouse model of HD, it increased neurogenesis in the striatum and cortex of HD mice, whilst also showing modest improvements in their behaviors.

### 1.4 miRNAs and proteins Amyotrophic Lateral Sclerosis

Amyotrophic lateral sclerosis (ALS) is one of the most common adult-onset NDs characterized by the progressive loss of somatic motor neurons in the spinal cord, resulting in progressive paralysis of muscular functions. In addition, dysphagia and dysarthria which are related to the degeneration of lower brain stem motor neurons may arise. Patients normally suffer from respiratory failure a few years into ALS resulting in death [3].

The most common genes associated with ALS are chromosome 9 open reading frame 72 (C9orf72), Superoxide dismutase (SOD1), TAR DNA-binding protein 43 (TARDBP), fused in sarcoma/translocated in liposarcoma (FUS/TLS) and matrin-3 [54]. Interestingly many ALS-linked genes, particularly TARDBP, FUS and matrin-3, are involved in RNA metabolism, including microRNA (miRNA) processing [[55], [56], [57]].

The importance of miRNA regulation in ALS was identified for the first time when different miRNA profiles were detected in ALS patients compared to healthy controls in the blood and cerebrospinal fluid (CSF), indicating that these small RNAs could be involved in the pathogenesis of ALS [[58], [59], [60]]. Interestingly, several miRNAs associated with maintenance of the central nervous system and cell death pathways were hampered in human samples isolated from the spinal cord of ALS patients [61].

In ALS, neuroinflammation is a major contributor to the disease through microglial activation, misregulation of immune-related genes, and recruitment of monocytes to affected tissues. Several miRNAs, such as let-7, miR-148 b-5p, miR-577, miR-133 b, miR-140-3p and miR-155 seem to be misregulated and involved in controlling translation of genes implicated in inflammatory pathways in the ALS context. For example, miR-155 promotes tissue inflammation by recruiting macrophages and increase of pro-inflammatory cytokine secretion by binding to suppressor of cytokine signaling 1 (SOCS1) mRNAs and recently levels of miR-155 in ALS human and mouse CSF has been shown to be significantly increased. In addition, anti-miR-155 is able to significantly enhance survival time of affected animals [[62], [63], [64]].

ALS has been linked with apoptosis and the ER stress response in motor neurons [65]. As mentioned above, defects in protein folding or degradation leads to increased accumulation of aggregated or misfolded proteins in the ER, which initiates the ER stress response and apoptosis. The ER stress-induced transcription factor Activating Transcription Factor-4 (ATF4) enhances expression of miR-29a and is correlated with reduced expression of myeloid leukemia cell differentiation protein (Mcl-1) [131], which is involved in the apoptosis pathway [65,66]. miR-29a levels increase during ER stress and elevated levels are also seen in the spinal cords of ALS mice at postnatal day 70 [65,67].

Under cellular stress mutant TDP-43 and FUS can interact with different proteins linked with RNA metabolism, leading to the development of protein aggregates and the formation of stress granules (SGs). It has been suggested that SGs could be precursor structures of the pathological protein inclusions observed in NDs [68]. Indeed, SGs assemble when eukaryotic translation initiation factor 2 alpha (eIF2α) is phosphorylated and this alteration is associated with neurotoxicity in ALS animal models [69]. SGs contain many RBPs prone to aggregation, such as TDP-43 and FUS, which are involved in miRNA metabolism [69]. TDP-43 interacts with both Drosha and the Dicer complexes, and FUS enhances miRNA production through Drosha [55,70]. Additionally, TDP-43 plays a key role in the post-transcriptional maturation of a subset of miRNAs and mislocalization of the TDP-43 protein in cytoplasmic aggregates seems to be associated with reduction in Drosha and Dicer processing of TDP-43-regulated miRNAs [55]. The impairment in miRNA biogenesis has been related to the stress response induced by mutations in ALS related genes, such as TDP-43, FUS, and SOD1. Overall, these findings suggest a potential link between defective miRNA biogenesis and ALS due to impaired Dicer processing.

A recent interesting finding demonstrated that misfolded proteins, such as ALS-linked variants of SOD1, accumulate and aggregate within SGs which decreases the dynamics of SGs, changes SG composition, and initiates an aberrant liquid-to-solid transition of *in vitro* reconstituted compartments [71]. Recruitment of chaperone proteins prevent the formation of aberrant SGs and promotes SG disassembly when the stress is removed. Although SGs do not necessarily contain miRNAs, they are tightly associated with mRNA processing bodies (PBs) which are heavily linked with sites of miRNA-mediated gene silencing and composed of aggregated mRNP complexes. SG clearance relies on nesprin-1-mediated microtubule contacts with PBs and PBs contain ALS related proteins, such as matrin-3, which shuttles from PBs to SGs during cellular stress [57,72,73]. Interestingly, nesprin-1 is able to interact with matrin-3 and both proteins are required for miRNA-mediated gene silencing. Furthermore, nesprin-1 mutations are associated with Ataxia and because matrin-3 mutations cause ALS these data suggest that hampered miRNA-mediated gene silencing are likely to cause ND [74,75].

## 2 Concluding remarks

Differential miRNA expression patterns observed in subjects suffering from NDs could represent disease signature and therefore be valuable for detecting the early onset of NDs and for development of new miRNA-based therapeutics. MiRNAs can be released as circulating molecules into bodily fluids such as blood, CSF and urine, which makes isolating samples for diagnosis easy and cheap. Furthermore, circulating miRNAs are usually bound to mRNP complexes and/or in exosomes, which increases their stability and makes them ideal biomarkers.

MiRNAs have the potential to be therapeutic molecules, where anti-mirs and sponges can be used to target pathological upregulated miRNAs and miRNA mimics target down-regulated ones. Although care would be needed as manipulating levels of active miRNAs using these methods may have profound affects on other signaling pathways which are needed to main neurological health. Delivery of these miRNA-manipulating tools will need to be carried out virally to proper cells and be able to cross the blood–brain barrier (BBB). Adeno-associated virus AAV9's ability of crossing the BBB after systemic administration opened new expectations for the development of gene therapy approaches for neurological disorders [76]. For example, this method has recently been used to investigate the therapeutic potential of developing AAV-mediated RNAi gene therapy for ALS [77]. One study was able to delay but not to prevent ALS progression and in another study AAV9 infected animals showed increased lifespan by 20% whilst preserving muscle strength and both motor and respiratory functions [78]. Therefore, these studies show that targeting NDs using miRNA-based therapy is plausible, however much work is needed to increase it efficacy.
